# MicroRNA-4639 Is a Regulator of DJ-1 Expression and a Potential Early Diagnostic Marker for Parkinson’s Disease

**DOI:** 10.3389/fnagi.2017.00232

**Published:** 2017-07-21

**Authors:** Yimeng Chen, Chao Gao, Qian Sun, Hong Pan, Pei Huang, Jianqing Ding, Shengdi Chen

**Affiliations:** ^1^Laboratory of Neurodegenerative Diseases, Institute of Health Sciences, Shanghai Institutes for Biological Sciences, Chinese Academy of Sciences, University of Chinese Academy of Sciences Shanghai, China; ^2^Department of Neurology and Institute of Neurology, Ruijin Hospital, Shanghai Jiao Tong University School of Medicine Shanghai, China; ^3^Key Laboratory of Stem Cell Biology, Institute of Health Sciences, Shanghai Institutes for Biological Sciences, Chinese Academy of Sciences, School of Medicine, Shanghai Jiao Tong University Shanghai, China; ^4^School of Biomedical Engineering and Med-X Research Institute, Shanghai Jiao Tong University Shanghai, China

**Keywords:** Parkinson’s disease, hsa-miR-4639-5p, plasma, biomarker, DJ-1, oxidative stress

## Abstract

Parkinson’s disease (PD) is the second most common neurodegenerative disorder and has profound impacts on the daily lives of patients. However, there is a lack of effective biomarkers for early diagnosis, and the mechanisms of PD pathogenesis remain obscure. microRNAs (miRNAs) are post-transcriptional gene regulators and can be easily detected in plasma, which suggests a promising role as diagnostic markers. Here, we aimed to explore a peripheral biomarker, which not only can be applied for early diagnosis of PD but also has the potential to be a therapeutic target. Through miRNA microarray screening and further validation in plasma from 169 sporadic PD patients, 170 healthy controls, and 60 essential tremor (ET) patients, hsa-miR-4639-5p level was identified to be significantly up-regulated in PD patients. Also, it was able to discriminate between early PD patients (disease duration ≤2 years or Hoehn and Yahr stage 1–2.5) and healthy controls. Furthermore, hsa-miR-4639-5p was shown to negatively regulate DJ-1 (PARK7), a well-known PD-related gene, in the post-transcriptional level. Abnormal up-regulation of hsa-miR-4639-5p caused down-regulation of DJ-1 protein level, leading to severe oxidative stress and neuronal death. In conclusion, hsa-miR-4639-5p has the potential to be a peripheral diagnostic biomarker and therapeutic target for early PD.

## Introduction

Parkinson’s disease (PD) is the second most common age-dependent neurodegenerative disorder. It affects approximately 1.7% of the population over 65 and has profound impacts on patients’ daily lives (Zhang et al., 2005; Wright Willis et al., 2010). However, there is a lack of effective biomarkers for early diagnosis of PD, and the molecular mechanisms of neurodegeneration is not completely understood.

PD is a chronic progressive neurodegenerative disease. Symptoms of PD occur 10–20 years after the beginning of neurodegeneration (Stocchi et al., 2015). Normally, dopaminergic neurons have already degenerated by over 60% when patients are diagnosed with PD in the clinic (Savitt et al., 2006). Therefore, patients miss the optimal period for anti-Parkinson medication treatment, especially disease-modifying therapies. It is highly imperative to explore biomarkers that allow for accurate diagnosis at the early stage of PD.

A combination of environmental and genetic factors are believed to account for the majority of PD cases (Klein and Schlossmacher, 2007). Several PD-associated genes have been identified, including SNCA, LRRK2, DJ-1 and others (Klein and Westenberger, 2012). Abnormal expressions of disease-related genes are believed to contribute to PD pathogenesis. MicroRNAs (miRNAs) play important roles in gene post-transcriptional regulation and have received much attention in recent years. They are small non-coding RNAs of approximately 20–24 nucleotides (nt) that conduct target mRNA degradation or translational inhibition through sequence complementarity to the 3′untranslated regions (3′UTR) of mRNAs (Ghildiyal and Zamore, 2009). Abnormal miRNA expression levels have been linked to several neurological disorders, including PD (Abe and Bonini, 2013; Heman-Ackah et al., 2013). miRNAs may contribute to PD pathogenesis by regulating disease-associated genes, allowing us to better understand the disease mechanisms. Also, miRNAs have the potential to be therapeutic targets for novel drug development.

Plasma has been widely used in biomarker studies because it is non-invasive and readily obtainable. In this study, we profiled plasma miRNA expressions in PD patients and healthy controls, and identified plasma hsa-miR-4639-5p to be the potential early diagnosis biomarker for PD. Moreover, DJ-1 (PARK7) was proved as a target gene of hsa-miR-4639-5p, interpreting the potential mechanism of dysregulated miRNA and PD pathogenesis.

## Materials and Methods

### Patients and Samples

In this study, 189 patients with PD, 60 patients with ET, and 190 healthy controls were recruited from the Department of Neurology, Ruijin Hospital, Shanghai Jiao Tong University School of Medicine from March 2014 to December 2015. The clinical diagnosis of PD was established by a senior movement disorder specialist using the clinical criteria for PD (the United Kingdom PD Society Brain Bank clinical diagnostic criteria; Calne et al., 1992; Hughes et al., 1992). PD symptoms were evaluated using the Unified Parkinson’s Disease Rating Scale (UPDRS; van Hilten et al., 1994) and the modified Hoehn and Yahr scale (Hoehn and Yahr, 1967). In this study, no family history of parkinsonism was present in all PD patients. None of them carries DJ-1 L166P mutation, which is associated with autosomal recessive early-onset PD (Bonifati et al., 2003), and confers reduced protein stability of DJ-1 (Moore et al., 2003). The control samples, including healthy persons and ET patients, were matched to PD patients in gender, age, ethnicity, genetically unrelated, and had no family history of parkinsonism. All the subjects were of Chinese Han ancestry. The study was approved by the Ethics Committee of Ruijin Hospital affiliated with Shanghai Jiaotong University School of Medicine and the written informed consent was obtained from all participants. All procedures performed in studies involving human participants were in accordance with the ethical standards of the institutional and/or national research committee and with the 1964 Helsinki Declaration and its later amendments or comparable ethical standards. The demographics and clinical characteristics of participants for biomarker study were summarized in Table 1.

**Table 1:** Summary of demographics and clinical characteristics of donors.

	PD	NC	ET
	*n* = 169	*n* = 170	*n* = 60
Gender			
Male, *n* (%)	81 (47.9%)	32 (53.3%)	83 (48.8%)
Female, *n* (%)	88 (52.1%)	28 (46.7%)	87 (51.2%)
Age (years)	61.9 ± 5.1	61.6 ± 3.3	61.5 ± 7.2
Age at onset (years)	56.1 ± 6.7	-	52.8 ± 12.0
Disease duration (years)	5.8 ± 4.2	-	8.7 ± 8.8
Hoehn and Yahr stage	2.0 ± 0.8	-	-
LDED (mg/day)	500.7 ± 350.5	-	-
UPDRS total	37.5 ± 18.2	-	-
UPDRS I	1.5 ± 1.6	-	-
UPDRS II	10.9 ± 4.9	-	-
UPDRS III	25.2 ± 13.1	-	-
MMSE score	26.7 ± 2.7	-	-

*Data are expressed as numbers with percentages in parentheses or as mean ± SD. LDED, levodopa daily equivalent dose; UPDRS, unified Parkinson’s disease rating scale; MMSE, Mini–Mental State Examination*.

Blood samples were collected in tubes containing EDTA. Plasma was separated within 4 h by centrifuging the blood at 3000× *g* for 10 min. Plasma was immediately separated into a 1.5 ml RNase-free centrifuge tube and stored at −80°C until RNA isolation. Three PD samples and five healthy control samples were selected for miRNA array analysis, and the remaining samples were saved for validation.

### MicroRNA Microarray

Plasma samples from PD patients and healthy controls were submitted to Kangchen Corporation (Kangchen, Shanghai, China) for miRNA microarray analysis. Total RNA was harvested from plasma using TRI reagent BD (MRCgene, TB-126) according to the manufacturer’s instructions. The RNA samples were labeled with the miRCURY™ Hy3™/Hy5™ Power Labeling Kit (Exiqon, Vedbaek, Denmark) and hybridized to the miRCURY™ LNA Array (v.18.0; Exiqon). miRNA expression data were normalized and chosen for differentially expressed miRNAs screening.

### Quantitative Real-Time PCR

Microarray results were confirmed by qRT-PCR. For miRNA analysis, total RNA was extracted from plasma with TRIzol LS reagent (Invitrogen) and cDNAs of specific miRNAs were synthesized with the specific reverse transcript primers. Delta delta CT method was used to calculate miRNA expression. Plasma miRNA expression results were normalized to the expression of endogenous miRNA hsa-miR-191-5p.

RT primer for hsa-miR-191-5p:
5′GTCGTATCCAGTGCGTGTCGTGGAGTCGGCAATTGCACTGGATACGACCAGCTG3′
RT primer for hsa-miR-4639-5p:
5′GTCGTATCCAGTGCGTGTCGTGGAGTCGGCAATTGCACTGGATACGACTCAATC3′
QPCR primers for hsa-miR-191-5p:
F: 5′GGCAACGGAATCCCAAAAG3′; R: 5′GTGCGTGTCGTGGAGTCG3′
QPCR primers for hsa-miR-4639-5p:
F: 5′GGGGTTGCTAAGTAGGCTGA3′; R: 5′GTGCGTGTCGTGGAGTCG3′

For DJ-1 mRNA analysis, total RNA was extracted from cells with TRIzol reagent (Invitrogen) and DJ-1 mRNA expression levels were normalized to GAPDH.

QPCR primers for human DJ-1:
F: 5′CATTCTCACTGTGTTCGCT3′; R: 5′TCCGCAAAAGTAGTAAGGAC3′
QPCR primers for human GAPDH:
F: 5′AGGGCTGCTTTTAACTCTGGT3′; R: 5′CCCCACTTGATTTTGGAGGGA3′

### Antibodies and Reagents

The following antibodies were used: rabbit polyclonal anti-PARK7/DJ-1 antibody (Abcam, ab18257, Cambridge, UK), mouse monoclonal anti-β-actin antibody (Sigma-Aldrich, Clone AC-15, St. Louis, MO, USA), horseradish peroxidase (HRP)-conjugated goat anti-mouse IgG, goat anti-rabbit IgG (Jackson ImmunoResearch Laboratories, PA, USA). 5-(and-6)-chloromethyl-2′,7′-dichlorodihydrofluorescein diacetate, acetylester (CM-H2DCFDA) reagents were purchased from Invitrogen (CA, USA). Dihydroethidium (DHE) reagents were purchased from Beyotime (Jiangsu, China). All chemicals were purchased from Sigma-Aldrich (St. Louis, MO, USA) unless otherwise stated.

### miRNA mimics and Inhibitors

miRNA mimics and inhibitors were purchased from Ribobio Company (Guangzhou, China). Universal negative controls for mimics and inhibitors were based on the sequences of *C. elegans*, which has been confirmed to have minimal homeology to all known miRNAs of miRBase 18.0. All of the sequences of miRNAs are from the Sanger miRNA database^1^.

### Cell Viability Detection by Cell Counting Kit-8 (CCK-8)

SH-SY5Y cells were seeded in a 96-well-plate at the number of 5000/well. Twenty-four hours after transfecting miRNA mimics/inhibitors, cells were treated with neurotoxic substance MPP^+^ (0.5 mM) or rotenone (0.15 μM) for another 24 h. Ten microliters of CCK-8 (Obio Technology, Shanghai) was added to the culture medium, and the viability of the cells was measured 4 h later at 450 nm with a Microplate reader (Synergy Mx, Bio-Tek, Winooski, VT, USA) according to the manufacturer’s instructions.

### Statistical Analysis

Data from three or more independent experiments are presented as the mean ± SD. Statistical analysis were done using SPSS 22.0 and GraphPad Prism 5.0 softwares. Student’s *t*-test, one-way analysis of variance (ANOVA), or Kruskal-Wallis test were used for statistical analysis. Correlations between miRNA levels and clinical characteristics were tested with Spearman’s rank correlation analysis. Differences with *p* < 0.05 were defined as the threshold for significance.

Information about the complete methods is provided in Supplementary Material. We used the same methodologies, such as cell culture and transfection, western blotting, dual-luciferase reporter assay and reactive oxygen species (ROS) measurement, as the ones employed in our previous study (Xiong et al., 2014).

## Results

### Plasma miRNA Expression Profiles in PD Patients and Healthy Controls

**Figure 1:**
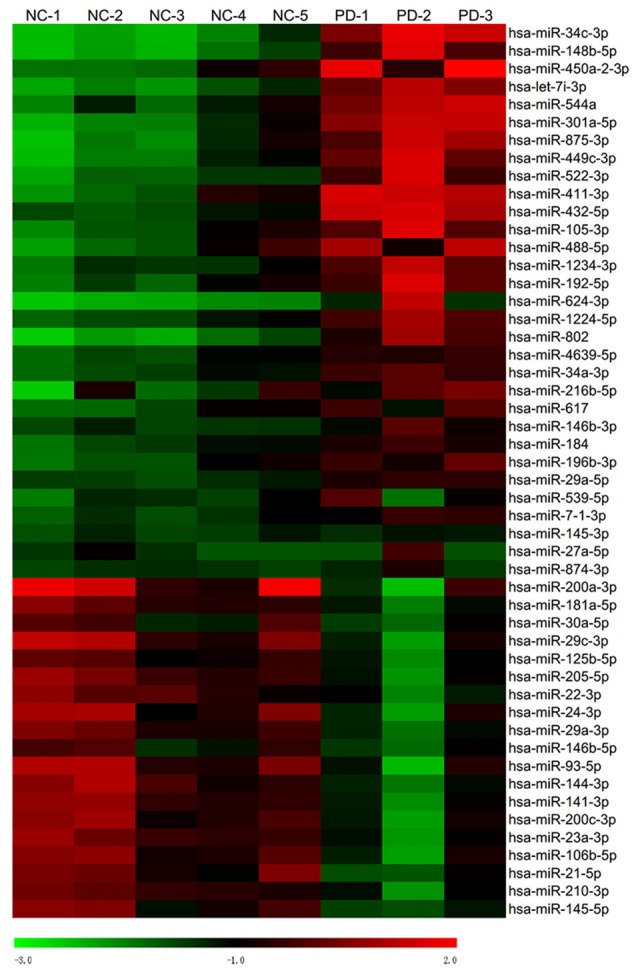
Heat map of miRNA microarray expression data of PD patients and healthy controls. Hierarchical clustering represented expression of plasma miRNAs of controls and PD patients. Five normal control samples and three PD samples of plasma were used for miRNA microarray analysis. Rows: miRNAs. Columns: control and PD samples. Red, black and green indicate the up-regulation, unchanged expression and down-regulation of miRNAs, respectively.

**Figure 2:**
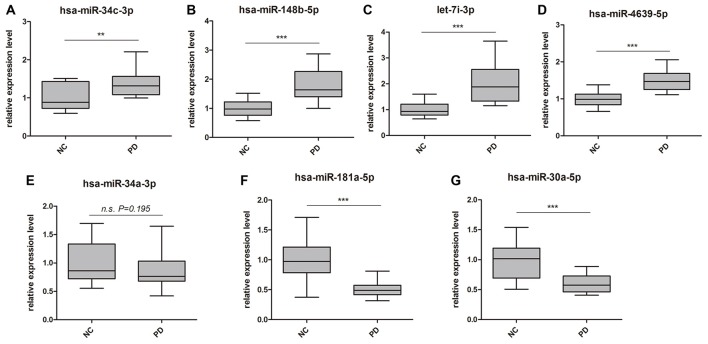
Validation of miRNA microarray by quantitative PCR (qPCR). Box-plots present distribution of normalized expression of **(A)** hsa-miR-34c-3p, **(B)** hsa-miR-148b-5p, **(C)** let-7i-3p, **(D)** hsa-miR-4639-5p, **(E)** hsa-miR-34a-3p, **(F)** hsa-miR-181a-5p and **(G)** hsa-miR-30a-5p between PD patients (*n* = 20) and healthy controls (*n* = 20). Among the seven miRNAs, which showed significantly change in microarray analysis, six of them were confirmed with qPCR. qPCR results were normalized to housekeeping hsa-miR-191-5p. **p < 0.01, ***p < 0.001, n.s., not significant.

Global plasma miRNA expression profiles were measured between the early PD group (Hoehn and Yahr stage 1–2.5) and healthy control group by miRNA microarray. The global miRNA expression patterns between the two groups were significantly different (Figure 1). Analysis of miRNA expression profiles showed 50 miRNAs were significantly dysregulated vs. controls, including 31 up-regulated miRNAs and 19 down-regulated miRNAs (fold change >1.5 or < −1.5, *P*-value <0.05; Figure 1). Next, seven miRNAs (hsa-miR-34c-3p, hsa-miR-148b-5p, hsa-let-7i-3p, hsa-miR-4639-5p, hsa-miR-34a-3p, hsa-miR-181a-5p and hsa-miR-30a-5p) were picked for validation with plasma samples from 20 sporadic PD patients and 20 healthy controls. The results showed similar expression tendency with that of microarray, except hsa-miR-34a-3p (Figure 2), indicating that microarray data was reliable in our study.

### hsa-miR-4639-5p was Predicted to Regulate DJ-1

To better understand the roles of observed dysregulated miRNAs and the underlying mechanism in PD pathogenesis, we utilized several prediction tools including Targetscan (Grimson et al., 2007), miRanda (Betel et al., 2008) and miRWalk (Dweep et al., 2011) to predict biological targets of above-mentioned miRNAs. Among a number of target gene candidates, we were particularly interested in PD-related genes, which have clear links with the pathogenesis of the disease. Notably, hsa-miR-4639-5p was predicted to relate to DJ-1 (PARK7), which plays a crucial role in cellular oxidative stress response (Shendelman et al., 2004). In PD patients, decreased DJ-1 protein levels have been observed in several brain areas (Kumaran et al., 2009; Nural et al., 2009; Miñones-Moyano et al., 2011). DJ-1 deficiency could lead to severe mitochondria fragmentation (Irrcher et al., 2010) and ROS burst, contributing to increased sensitivity to oxidative stress and apoptosis (Demasi and Davies, 2003). Based on these studies and our prediction data, we wondered if hsa-miR-4639-5p indeed participates in the regulation of DJ-1 expression. Thus, hsa-miR-4639-5p was chosen for further study.

### hsa-miR-4639-5p Post-Transcriptionally Regulate DJ-1 Expression via its 3′UTR

**Figure 3:**
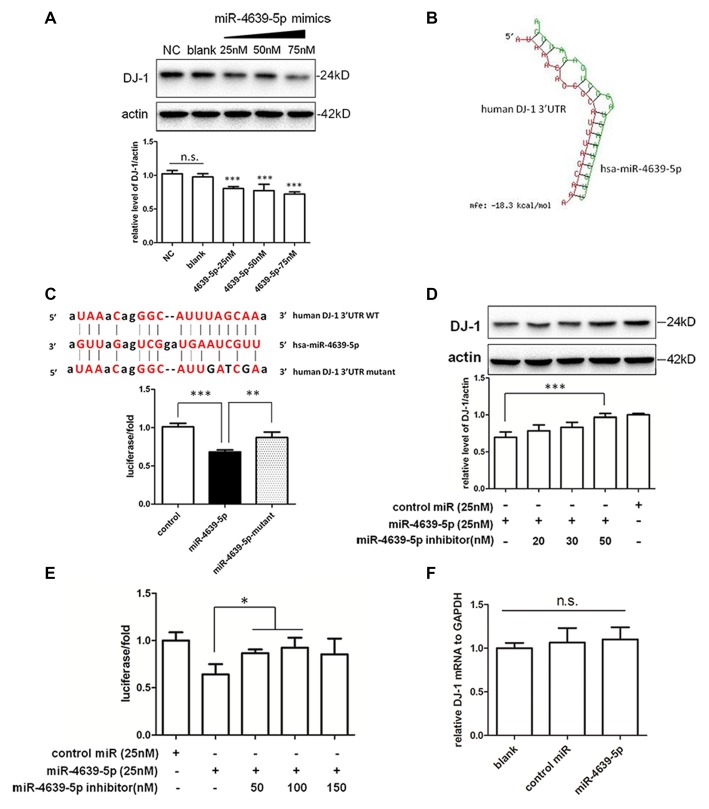
Identification of hsa-miR-4639-5p as regulator of DJ-1 expression.** (A)** Western blotting indicated that endogenous DJ-1 expression in HEK-293T cells was down-regulated by transfection of hsa-miR-4639-5p mimics in a dose-dependent manner. **(B)** RNA duplex formation of hsa-miR-4639-5p and human DJ-1 3′UTR sequence predicted by RNAhybrid. **(C)** Sequence of wild-type (WT) and mutated (mutant) binding site of hsa-miR-4639-5p within human DJ-1 3′UTR. The ratio of Renilla luciferase activity to firefly luciferase activity is shown. **(D)** DJ-1 protein level was restored in hsa-miR-4639-5p inhibitors-transfected HEK-293T cells in a dose-dependent manner. **(E)** Luciferase assay. Transfection of hsa-miR-4639-5p inhibitors could restore relative luciferase activity in a dose-dependent manner. **(F)** hsa-miR-4639-5p overexpressed cells had no alternation in DJ-1 mRNA. Data shown as mean ± SD of at least three independent experiments. One-way analysis of variance (ANOVA) was used for comparison between multiple groups followed by Bonferroni *post hoc* correction. *P < 0.05, **p < 0.01, ***p < 0.001, n.s., not significant.

In our preliminary data, hsa-miR-4639-5p was observed to be abnormally up-regulated in PD patients. In order to mimic such pathological change, hsa-miR-4639-5p was overexpressed by the transient transfection of hsa-miR-4639-5p mimics, and the level of DJ-1 protein was analyzed by western blotting. HEK-293T cells were used in experiments, which express significant amounts of endogenous DJ-1. Overexpression of hsa-miR-4639-5p resulted in a dose-dependent decrease in DJ-1 protein level (Figure 3A). Luciferase assay was used to further determine whether DJ-1 is a putative target for hsa-miR-4639-5p. Luciferase reporter construct was generated with the 3′UTR of human DJ-1 mRNA cloned after Renilla luciferase coding sequence. The construct was co-transfected with hsa-miR-4639-5p mimics or negative control. Accordingly, hsa-miR-4639-5p was observed to repress the reporter activity markedly (Figure 3C, black bar). These results suggest that hsa-miR-4639-5p might be the human DJ-1 expression regulator.

Seed sequence at nucleotides 2–8 of the 5′ end of miRNA is fundamental for target recognition and binding (Bartel, 2009). We found that the seed sequences of hsa-miR-4639-5p are perfectly complementary to nucleotides 217–224 of human DJ-1 3′UTR. RNAhybrid (Krüger and Rehmsmeier, 2006), which is designed for miRNA target prediction, was used to test our result. The minimum free energy required for interaction between hsa-miR-4639-5p and the corresponding DJ-1 3′UTR binding site is −18.3 kcal/mol according to RNAhybrid calculation (Figure 3B). To confirm direct binding of hsa-miR-4639-5p to DJ-1 3′UTR, predicted binding sites were mutagenized as shown in Figure 3C. hsa-miR-4639-5p was not able to repress luciferase activity when mutagenized 3′UTR was applied, indicating that the predicted sequence is indeed the genuine binding site for hsa-miR-4639-5p.

To further prove the effects of abnormal up-regulated hsa-miR-4639-5p on DJ-1 expression, miRNA inhibitors (synthetic single-stranded anti-sense oligonucleotides designed to inhibit miRNA activity) were co-transfected with hsa-miR-4639-5p mimics into HEK-293T cells. hsa-miR-4639-5p inhibitors resulted in restored DJ-1 expression in a dose-dependent manner (Figure 3D). Luciferase activity of human DJ-1 was also significantly restored in the presence of hsa-miR-4639-5p inhibitors (Figure 3E). Meanwhile, alteration of DJ-1 protein levels by transfecting hsa-miR-4639-5p mimics or inhibitors was confirmed in human neuroblastoma SH-SY5Y cells (Supplementary Figure S1).

Furthermore, there was no significant change of the levels of DJ-1 mRNA in the cells transfected with hsa-miR-4639-5p mimics (Figure 3F), indicating that hsa-miR-4639-5p repressed DJ-1 protein level through affecting translation of DJ-1 mRNA.

### ROS Production and Cell Death Were Increased in miR-4639-5p-Overexpressed Cells

**Figure 4:**
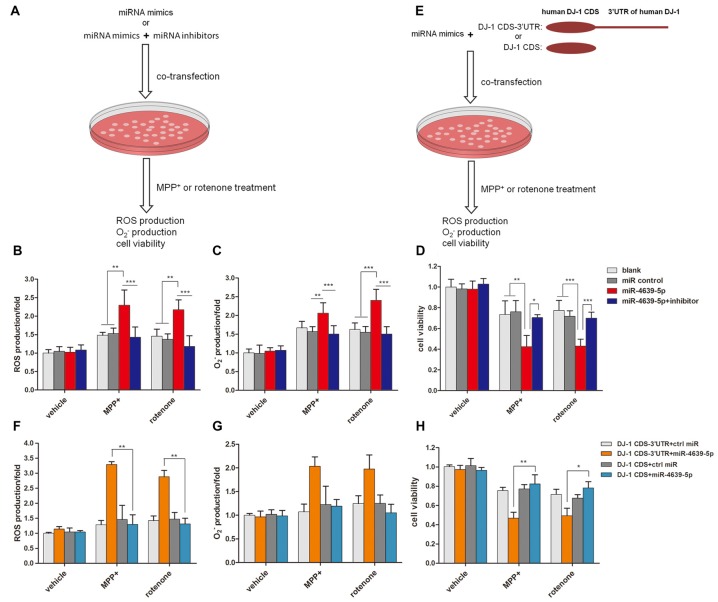
hsa-miR-4639-5p rendered cells more susceptible to oxidative stress.** (A)** Fifty nanomolar hsa-miR-4639-5p mimics were transfected into SH-SY5Y cells to over-express hsa-miR-4639-5p. Fifty nanomolar hsa-miR-4639-5p mimics and 50 nM hsa-miR-4639-5p-specific inhibitors were co-transfected to conduct loss-of-function experiments. Twenty-four hours after transfection, cells were treated with 0.5 mM MPP^+^ or 0.15 μM rotenone. Reactive oxygen species (ROS) production, ${\text{O}}_{\text{2}}^{-}$ production and cell viability were measured 24 h after neurotoxin treatment.** (B)** ROS production was measured by CM-H2DCFDA probe.** (C)** Superoxide level was measured by dihydroethidium (DHE) probe. The results showed significant difference between hsa-miR-4639-5p overexpressed cells and control cells. **(D)** Cell viability was measured by CCK-8. It revealed that hsa-miR-4639-5p overexpressed cells were more susceptible to MPP^+^ or rotenone insults. **(E)** Fifty nanomolar hsa-miR-4639-5p mimics and exogenous DJ-1 plasmids were co-transfected into SH-SY5Y cells. Twnenty-four hours after transfection, cells were treated with 0.5 mM MPP^+^ or 0.15 μM rotenone. ROS production, ${\text{O}}_{\text{2}}^{-}$ production and cell viability were measured 24 h after neurotoxin treatment. **(F,G)** Human DJ-1 with/without-3′UTR was re-introduced into cells. ROS and ${\text{O}}_{\text{2}}^{-}$ production upon MPP^+^ and rotenone treatment was compared between DJ-1CDS-3′UTR co-transfected cells and DJ-1CDS co-transfected cells. **(H)** Cell viability was compared between DJ-1CDS co-transfected cells and DJ-1CDS-3′UTR co-transfected cells. Data shown as mean ± SD of three independent experiments. One-way ANOVA was used for comparison between multiple groups followed by Bonferroni *post hoc* correction. *p < 0.05, **p < 0.01, ***p < 0.001.

Our previous study (Wang et al., 2011) suggested that DJ-1 is able to protect cells against oxidative insult. DJ-1 knock-down cells treated with MPP^+^ produced more ROS and were more vulnerable to oxidative stress than those of normal controls. Here, we investigated the effect of abnormal up-regulated hsa-miR-4639-5p on cellular anti-oxidative function when cells are exposed to stressful attacks. SH-SY5Y cells were transiently transfected by 50 nM miRNA mimics or 50 nM miRNA mimics + 50 nM miRNA inhibitors, followed by MPP^+^ or rotenone treatment (both of which are toxic substrates that induce oxidative stress). Twenty-four hours later, ROS levels and cell viability were measured (Figure 4A). The results showed that hsa-miR-4639-5p-overexpressed SH-SY5Y cells elicited more ROS and ${\text{O}}_{\text{2}}^{-}$ than the control cells or miRNA inhibitors co-transfected cells (Figures 4B,C). Moreover, cell viability decreased in hsa-miR-4639-5p-overexpressed cells compared to control cells or miRNA inhibitors co-transfected cells (Figure 4D).

To further investigate whether abnormal up-regulated hsa-miR-4639-5p impaired anti-oxidative function through its direct regulation on DJ-1 expression, DJ-1 protein was re-introduced into hsa-miR-4639-5p-overexpressed cells. We constructed two plasmids that included or did not include 3′UTR of human DJ-1 gene, namely, DJ-1CDS-3′UTR and DJ-1CDS. miRNA mimics + DJ-1CDS-3′UTR plasmid or miRNA mimics + DJ-1CDS plasmid were co-transfected into cells (Figure 4E). hsa-miR-4639-5p was able to inhibit exogenous DJ-1CDS expression when it’s 3′UTR was existed. However, it could not inhibit exogenous DJ-1 expression when only DJ-1CDS was transfected. The results showed that ROS production and cell death decreased in DJ-1CDS co-transfected cells compared to DJ-1CDS-3′UTR co-transfected cells (Figures 4F–H).

These results suggested that cells with abnormal up-regulated hsa-miR-4639-5p were more susceptible to MPP^+^ or rotenone-induced cell death as a consequence of DJ-1 depletion.

### Evaluation of the Early Diagnostic Potential of hsa-miR-4639-5p for PD

**Figure 5:**
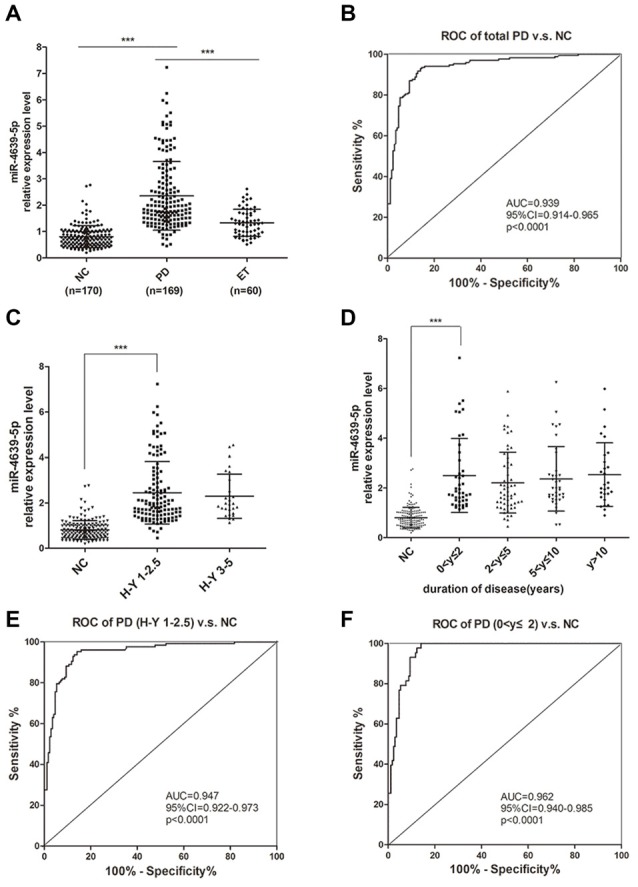
Plasma hsa-miR-4639-5p may serve as a biomarker for early diagnosis of PD.** (A)** Relative expression level of plasma hsa-miR-4639-5p was measured in total 170 normal controls, 169 PD patients and 60 ET patients.** (B)** Area under the receiver operating characteristic (ROC) curve is illustrated for hsa-miR-4639-5p level between 169 PD patients and 170 normal controls. Sensitivity = 93.49%, specificity = 85.29%. **(C)** hsa-miR-4639-5p level was significantly up-regulated in early stage of PD patients (Hoehn and Yahr stage 1–2.5) compared to normal controls. **(D)** hsa-miR-4639-5p level was significantly up-regulated in PD patients within two years from onset and maintains relatively high level during PD progression. **(E)** Area under the ROC curve is illustrated for hsa-miR-4639-5p level between 127 early PD patients (Hoehn and Yahr stage 1–2.5) and 170 normal controls. Sensitivity = 95.28%, specificity = 85.88%. **(F)** Area under the ROC curve is illustrated for hsa-miR-4639-5p level between 43 PD patients (disease duration ≤2 years) and 170 normal controls. Sensitivity = 100.00%, specificity = 85.88%. Data shown as mean ± SD. Kruskal-Wallis test was used for multiple comparisons among groups followed by Bonferroni *post hoc* correction. ***P < 0.001.

To further confirm the abnormal up-regulation of hsa-miR-4639-5p level in PD patients, we performed qPCR analysis of plasma hsa-miR-4639-5p level in a relatively large cohort of patients with PD (*n* = 169), healthy controls (*n* = 170) and patients with ET (*n* = 60). The demographics information of all participants is summarized in Table 1. The relative hsa-miR-4639-5p level in PD patients was significantly higher (*P* < 0.001) than that in healthy controls and ET patients (Figure 5A). Receiver operating characteristic (ROC) curve reflects a separation between the PD and normal subjects, with area under the curve (AUC) values up to 0.939 (95% CI = 0.914–0.965, sensitivity = 93.49%, specificity = 85.29%, Figure 5B).

Further, we wondered whether plasma hsa-miR-4639-5p level could serve as an early diagnosis marker for PD. Thus, PD patients were divided into sub-groups according to Hoehn and Yahr stage and disease duration, and stratified analysis was performed. Plasma hsa-miR-4639-5p level was found to be significantly up-regulated in early stage of PD and maintained relatively high levels during PD progression (Figures 5C,D). ROC curve showed that hsa-miR-4639-5p has adequate sensitivity and specificity to discriminate between early PD patients and healthy controls (PD patients with H-Y 1–2.5 vs. NC: sensitivity = 95.28%, specificity = 85.88%; PD patients with disease duration ≤2 years vs. NC: sensitivity = 100.00%, specificity = 85.88%; Figures 5E,F). Moreover, plasma hsa-miR-4639-5p level was uncorrelated with gender, age of disease onset, levodopa daily equivalent dose (LDED), and UPDRS score of PD patients (Supplementary Figure S2). Therefore, the elevated plasma level of hsa-miR-4639-5p in PD patients is stable.

## Discussion

In this study, we revealed a significantly up-regulated plasma hsa-miR-4639-5p level in PD patients. Stratified analysis demonstrated that hsa-miR-4639-5p was able to distinguish early PD patients (Hoehn and Yahr stage 1–2.5 or disease duration ≤2 years) from healthy people, indicating the promising potential of hsa-miR-4639-5p in early PD diagnosis.

Plasma, a non-invasive sample source, is easily accessed compared to brain samples or cerebrospinal fluid (CSF), which has been widely used in biomarker studies. miRNAs are small molecules with good chances to cross blood-brain barrier (Sun et al., 2012) and be secreted into body fluids from the central nervous system (CNS). They exhibit high stability in plasma, serum, urine and saliva (Chen et al., 2008), and can be detected as cell-free or exosome-derived molecules (Mitchell et al., 2008). Recent studies have shown that in CNS diseases such as stroke, Alzheimer’s disease (AD) and glioblastoma, the pathological processes in the brain could be reflected in peripheral miRNA expression patterns (Zeng et al., 2011; Ilhan-Mutlu et al., 2012; Rao et al., 2013; Wang et al., 2015). Thus, plasma miRNA expression levels in PD patients may help us understand the pathological processes in the brains as those aforementioned diseases. Moreover, our preliminary data supported the hypothesis that abnormally increased hsa-miR-4639-5p that is detected in plasma mainly comes from CNS-derived exosomes (data not shown), indicating that the peripheral miRNA level might reflect the pathophysiology of PD.

PD is a progressive neurodegenerative disease. Dopaminergic neurons may have degenerated by over 60% and synaptic function reduced by up to 80% before clinical diagnosis of PD is made (de la Fuente-Fernández et al., 2011). Developing biomarkers, which can predict early onset of the disease, can help design treatments to slow down disease progression. In this study, hsa-miR-4639-5p was selected from miRNA microarray as up-regulated miRNA in PD patients. Significantly high hsa-miR-4639-5p level in PD patients was affirmed in a relatively large cohort by qPCR validation (169 PD, 170 NC, *P* < 0.001). Even in the very beginning stage of the disease, most PD patients can be distinguished from healthy individuals by plasma hsa-miR-4639-5p level. Therefore, hsa-miR-4639-5p has the potential to be an early diagnostic marker for PD. Further, our findings will be more convincing if up-regulation of hsa-miR-4639-5p could be verified in prodromal PD patients. Future work is still needed before hsa-miR-4639-5p can actually become a biomarker for PD early diagnosis.

As one of the causal genes identified for autosomal recessive PD, DJ-1 (PARK7) is believed to have an important protective role against oxidative insult in the brain (Shendelman et al., 2004). In familial PD, loss-of-function-mutation (L166P) in DJ-1 reduces protein stability, leading to the lower level of DJ-1 and impaired anti-oxidative function (Moore et al., 2003; Wang et al., 2011). Meanwhile, decreased DJ-1 protein level was also observed in sporadic PD cases (Kumaran et al., 2009; Nural et al., 2009; Miñones-Moyano et al., 2011), but the molecular mechanisms are not fully understood. Here, we discovered for the first time that human DJ-1 could be post-transcriptional regulated by miRNA. hsa-miR-4639-5p suppressed the expression of human DJ-1 through conserved binding to the 3′-UTR of DJ-1 mRNA, causing down-regulation of DJ-1 protein level, leading to severe oxidative stress and neuronal death.

hsa-miR-4639-5p was first identified by next-generation sequencing in 2011 (Persson et al., 2011). As a newly discovered miRNA, the molecular function and potential target genes of hsa-miR-4639-5p were mostly unknown. Moreover, there is no corresponding mouse mmu-miR-4639 reported in miRBase. Thus, we did *in vitro* studies to confirm the regulation of hsa-miR-4639-5p on DJ-1 expression in human cell lines (HEK-293T and SH-SY5Y). hsa-miR-4639-5p is expressed in both peripheral blood and CNS (Supplementary Figure S3). Our preliminary data suggested that increased plasma miR-4639-5p in PD patients mainly comes from CNS-derived exosomes (L1CAM^(+)^ exosomes, data not shown). It supported the idea that abnormal increased miR-4639-5p could be detected in plasma, and its inhibition on DJ-1 expression happened in brain. As a peripheral biomarker, plasma hsa-miR-4639-5p may reflect pathology in PD brains. Thus, an interesting extension of present study would be to investigate hsa-miR-4639-5p expression in PD brain samples. In addition, the mechanisms contributing to up-regulation of hsa-miR-4639-5p remain obscure. In our opinion, the dysregulation of hsa-miR-4639-5p in PD patients may be due to abnormal transcriptional regulation or an aberrated miRNA maturation process. Future studies are needed to explore the underlying mechanisms.

In conclusion, we identified plasma hsa-miR-4639-5p that could distinguish PD patients from healthy controls, which has the potential to be an early diagnosis biomarker for sporadic PD. Meanwhile, our results revealed the regulation mechanism of hsa-miR-4639-5p on human DJ-1 expression, indicating that the modulation of hsa-miR-4639-5p may be a therapeutic strategy for PD.

## Author Contributions

SC designed and supervised the whole study, involved in manuscript preparation and patients’ diagnoses. JD designed and supervised the study, and was responsible for manuscript preparation and data analysis. YC was responsible for the acquisition, analysis of data for the work and wrote the first draft of the manuscript. CG was involved in the data analysis and manuscript preparation. QS and PH recruited participants and collected plasma samples. HP predicted miRNA-targets using bioinformatics and analyzed the data.

## Conflict of Interest Statement

The authors declare that the research was conducted in the absence of any commercial or financial relationships that could be construed as a potential conflict of interest.

## Footnotes

^1^http://microrna.sanger.ac.uk/sequences

## Abbreviations

AD: Alzheimer’s disease
CNS: central nervous system
ET: essential tremor
miRNA: microRNA
MPP^+^: 1-methyl-4-phenylpyridinium
${\text{O}}_{\text{2}}^{-}$: superoxide
PD: Parkinson’s disease
ROS: reactive oxygen species
UTR: untranslated region.
