# Supplementary material for: MicroRNA-4639 Is a Regulator of DJ-1 Expression and a Potential Early Diagnostic Marker for Parkinson’s Disease
Source: Front Aging Neurosci. 2017 Jul 21;9:232. doi: 10.3389/fnagi.2017.00232 (PMC5519626; doi:10.3389/fnagi.2017.00232)
Supplement: Supplementary file 1 [file Data_Sheet_1.pdf]

Supplementary Material

MicroRNA-4639 is a regulator of DJ-1 expression and a potential early diagnostic marker for Parkinson's disease

Yimeng Chen[123], Chao Gao[24], Qian Sun[2], Hong Pan[123], Pei Huang[2], Jianqing Ding[2]* and Shengdi Chen[123]*

*** Correspondence:**
Shengdi Chen: chen_sd@medmail.com.cn and Jianqing Ding: jqding18@yahoo.com

1 Supplementary Methods

1.1 Cell culture and transfection

HEK-293T and SH-SY5Y cells were purchased from American Type Culture Collection, and maintained in Dulbecco's modified Eagle's medium (DMEM) with 10% fetal bovine serum and 100 U/mL penicillin/streptomycin. All the culture materials were purchased from Invitrogen. Cells were transfected with lipofectamine3000 (Invitrogen) according to the manufacturer's instruction.

1.2 Plasmid construction

Human DJ-1 cDNA was amplified from SH-SY5Y cell line, and ligated to pcDNA3.1 vector. For luciferase reporter assay, 3'UTR of human DJ-1 mRNA was amplified and cloned into psiCHECK-2 vector (Promega, WI, USA). Site-specific mutants were generated by a polymerase chain reaction (PCR) based site mutagenesis method in psiCHECK-2 vector.

1.3 Reactive Oxygen Species Measurement

Intracellular ROS production was measured by 5-(and-6)-chloromethyl- 2',7'-dichlorodihydrofluorescein diacetate (CM-H2DCFDA) assay. General superoxide level was measured by dihydroethidium (DHE) according to the manufacturer's instructions. SH-SY5Y cells were seeded in a 12-well-plate at the number of 60000/well. 24 hours after transfecting miRNA mimics/inhibitors, cells were treated with neurotoxic substance

MPP^+(0.5mM) or rotenone(0.15μM) for another 24 hours. Cells were incubated with 10μM CM-H2DCFDA or 5μM DHE probes at 37℃ for 30 minutes. The probe was then removed, and cells were washed twice using pre-warmed PBS to avoid background fluorescence brought by unloaded probe. The fluorescence was measured with a Microplate reader (Synergy Mx, Bio-Tek, America). For CM-H2DCFDA, the excitation was 485 nm and emission was 530 nm. For DHE, the excitation was 544 nm and emission was 612 nm.

1.4 Western blotting

For western blotting, HEK-293T and SH-SY5Y cells were lysed in Radio-Immunoprecipitation Assay (RIPA) buffer (50 mmol/L Tris HCl (pH 8.0), 150 mmol/L NaCl, 1% NP-40, 0.5% sodium deoxycholate, and 0.1% sodium dodecyl sulfate [SDS]) with protease inhibitor cocktail (Roche). Lysates (20 mg) were boiled in 4×SDS loading buffer, and the samples were separated by SDS-PAGE, transferred to a PVDF membrane, and detected by immunoblotting analysis with the indicated antibodies using Immobilon Western Chemiluminescent HRP Substrate (Millipore). All of the experiments were performed at least 3 times, and the most representative results were shown.

1.5 Luciferase assay

HEK-293T and SH-SY5Y cells were seeded in the wells of a 12-well plate. Cells were transfected with a mixture of luciferase reporter plasmids and miRNA mimics. 24 hours after transfection, luciferase activity was assayed on a Microplate reader (Synergy Mx, Bio-Tek, America) using the Dual-Luciferase Reporter Assay System (Promega) according to the manufacturer's protocol. The ratio of Renilla luciferase activity to firefly luciferase activity was calculated for each well.

2 Supplementary Figures

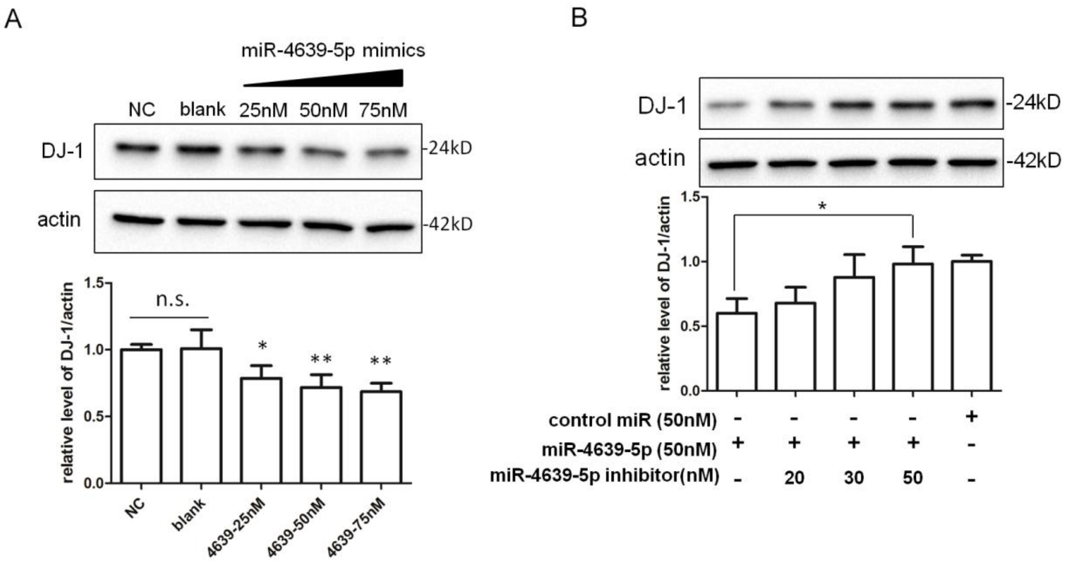

Supplementary Figure 1. Alteration of miRNA changed DJ-1 protein expression in SH-SY5Y cells.

(A) Western blotting indicated that endogenous DJ-1 expression in SH-SY5Y cells was down-regulated by transfection of hsa-miR-4639-5p mimics in a dose-dependent manner. Bar graph indicates the statistical analysis (Mean ± SD) of three independent experiments. **(B)** DJ-1 protein level was restored in hsa-miR-4639-5p inhibitor transfected SH-SY5Y cells in a dose-dependent manner. Data shown as mean±SD of at least three independent experiments. *p<0.05, **p<0.01, n.s., not significant.

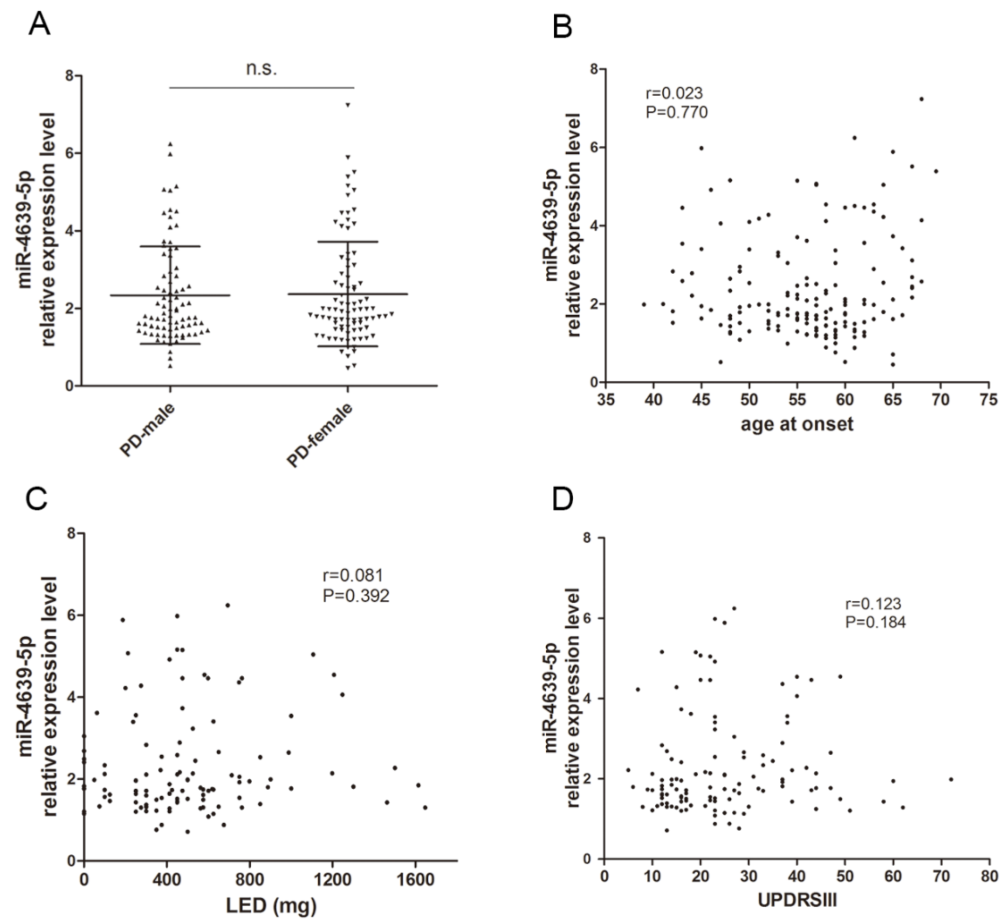

Supplementary Figure 2. Correlation between plasma hsa-miR-4639-5p level and clinical characteristics of PD patients.

(A) Relative plasma hsa-miR-4639-5p level had no significant difference between male and female PD patients. **(B)** No correlation between plasma hsa-miR-4639-5p level and PD onset age. **(C)** No correlation between plasma hsa-miR-4639-5p level and levodopa daily equivalent dose (LED) of PD patients. **(D)** No correlation between plasma hsa-miR-4639-5p level and unified Parkinson's disease rating scale (UPDRSⅢ) score of PD patients. Correlations were tested with Spearman’s rank correlation analysis. Data shown as mean±SD, n.s., not significant.

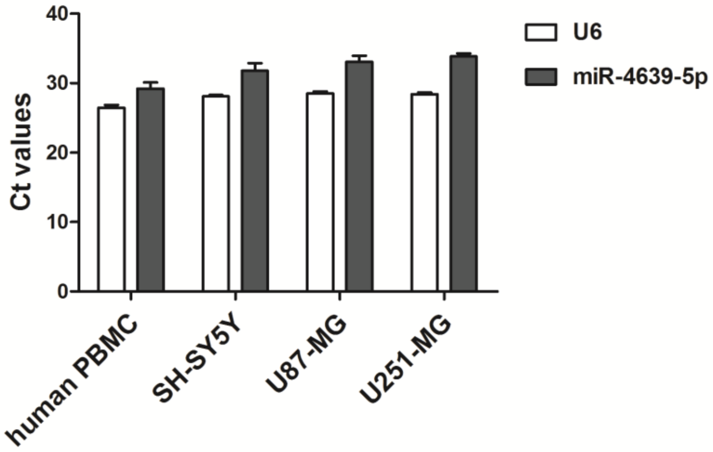

Supplementary Figure 3. hsa-miR-4639-5p is expressed in both peripheral blood and central nervous system.

Ct values of hsa-miR-4639-5p compared to housekeeping U6 in human peripheral blood mononuclear cells (PBMC), neuroblastoma SH-SY5Y, glioblastoma U-87 MG and U-251 MG cells were shown.
